# A *Klebsiella variicola* Plasmid Confers Hypermucoviscosity-Like Phenotype and Alters Capsule Production and Virulence

**DOI:** 10.3389/fmicb.2020.579612

**Published:** 2020-12-16

**Authors:** Nadia Rodríguez-Medina, Esperanza Martínez-Romero, Miguel Angel De la Cruz, Miguel Angel Ares, Humberto Valdovinos-Torres, Jesús Silva-Sánchez, Luis Lozano-Aguirre, Jesús Martínez-Barnetche, Veronica Andrade, Ulises Garza-Ramos

**Affiliations:** ^1^Laboratorio de Resistencia Bacteriana, Centro de Investigación Sobre Enfermedades Infecciosas, Instituto Nacional de Salud Pública, Cuernavaca, Mexico; ^2^Programa de Doctorado en Ciencias Biomédicas, Universidad Nacional Autónoma de México, México City, Mexico; ^3^Centro de Ciencias Genómicas, Universidad Nacional Autónoma de México, Cuernavaca, Mexico; ^4^Unidad de Investigación Médica en Enfermedades Infecciosas y Parasitarias, Hospital de Pediatría, Centro Médico Nacional Siglo XXI, Instituto Mexicano del Seguro Social, México City, Mexico; ^5^Departamento de Inmunología, Instituto Nacional de Salud Pública, CISEI, Cuernavaca, Mexico; ^6^Centro de Ciencias Genómicas, Laboratorio de Genómica Evolutiva, Universidad Nacional Autónoma de México, Cuernavaca, Mexico; ^7^Hospital Regional Centenario de la Revolución Mexicana, ISSSTE, Emiliano Zapata, Mexico

**Keywords:** Hypermucoviscosity, Plasmid curing, mating, virulence, Capsule production

## Abstract

Hypermucoviscosity (hmv) is a capsule-associated phenotype usually linked with hypervirulent *Klebsiella pneumoniae* strains. The key components of this phenotype are the RmpADC proteins contained in non-transmissible plasmids identified and studied in *K. pneumoniae*. *Klebsiella variicola* is closely related to *K. pneumoniae* and recently has been identified as an emergent human pathogen. *K. variicol*a normally contains plasmids, some of them carrying antibiotic resistance and virulence genes. Previously, we described a *K. variicola* clinical isolate showing an hmv-like phenotype that harbors a 343-kb pKV8917 plasmid. Here, we investigated whether pKV8917 plasmid carried by *K. variicola* 8917 is linked with the hmv-like phenotype and its contribution to virulence. We found that curing the 343-kb pKV8917 plasmid caused the loss of hmv, a reduction in capsular polysaccharide (*P* < 0.001) and virulence. In addition, pKV8917 was successfully transferred to *Escherichia coli* and *K. variicola* strains via conjugation. Notably, when pKV8917 was transferred to *K. variicola*, the transconjugants displayed an hmv-like phenotype, and capsule production and virulence increased; these phenotypes were not observed in the *E. coli* transconjugants. These data suggest that the pKV8917 plasmid carries novel hmv and capsule determinants. Whole-plasmid sequencing and analysis revealed that pKV8917 does not contain *rmpADC*/*rmpA2* genes; thus, an alternative mechanism was searched. The 343-kb plasmid contains an IncFIB backbone and shares a region of ∼150 kb with a 99% identity and 49% coverage with a virulence plasmid from hypervirulent *K. variicola* and multidrug-resistant *K. pneumoniae*. The pKV8917-unique region harbors a cellulose biosynthesis cluster (*bcs*), fructose- and sucrose-specific (*fru/scr*) phosphotransferase systems, and the transcriptional regulators *araC* and *iclR*, respectively, involved in membrane permeability. The hmv-like phenotype has been identified more frequently, and recent evidence supports the existence of *rmpADC*/*rmpA2*-independent hmv-like pathways in this bacterial genus.

## Introduction

Hypermucoviscosity (hmv) is a distinctive trait displayed by hypervirulent *Klebsiella pneumoniae* (hvKpn), and it is visualized by the formation of a viscous filament >5 mm by strains grown on agar plates. However, quantitative tests are recommended to distinguish hmv from non-hmv strains (Walker and Miller, 2020). A recent study showed that capsule and hmv are not the same process; hence, the capsule serves as a scaffold for hmv (Walker et al., 2019). The molecular mechanism underlying hmv in hvKpn strains involves multiple global regulators acting directly or indirectly on the cps region (Ares et al., 2016; Walker and Miller, 2020). Among the set of proposed regulators, the *rmpADC* locus is highly correlated with hmv in hvKpn (Walker et al., 2019; Walker et al., 2020). However, it is not well known whether hmv could contribute to a higher virulence level when hypervirulence traits are absent. Different works have reported *K. pneumoniae, K. variicola* and *K. quasipneumoniae* isolates displaying the hmv-like phenotype as the genes involved are unknown and apparently not related to *rmpADC* (Yu et al., 2006; Li et al., 2014; Arena et al., 2015; Cubero et al., 2016; Garza-Ramos et al., 2016; Harada et al., 2019; Imai et al., 2019). Consequently, this scenario suggests the existence of *rmpADC*/*rmpA2-*independent hmv-like pathways in *Klebsiella*.

*Klebsiella variicola* has gained clinical recognition as an emerging human pathogen not only for its capacity to acquire antimicrobial resistance and virulence genes but also for being widespread and types of infections (Rodriguez-Medina et al., 2019). We previously described the first *K. variicola* clinical isolate categorized as hypermucoviscous that harbors one 343-kb plasmid (Garza-Ramos et al., 2015; Rodriguez-Medina et al., 2019). The present study focuses on the genetic analysis of the pKV8917 plasmid as a determinant capable of transfer hmv-like and its role in virulence.

## Materials and Methods

### Bacterial Isolate Identification

*Klebsiella variicola* 8917 clinical isolate was previously reported (Garza-Ramos et al., 2015). Briefly, the isolate was obtained from the sputum of an elderly male patient at the Hospital Regional Centenario de la Revolución Mexicana in Morelos, Mexico, in 2011.

### Conjugation Assays, *in vitro* Induction of Nalidixic Acid-Resistant *Klebsiella variicola* Mutants and Plasmid Stability

The non-hmv *K. variicola* F2R9 (DSM 15968, ATCC BAA 830) nalidixic acid-resistant and *Escherichia coli* J53-2 rifampin-resistant were used as recipient strains. The conjugation experiments were carried out in broth. *In vitro* nalidixic acid resistance was induced in *K. variicola* F2R9 because this strain is susceptible to all antibiotic families (except ampicillin). A nalidixic acid-resistant mutant was obtained from a serial passage of the susceptible strain in a medium with increasing concentrations of nalidixic acid (from 16 to 128 μg ml^–1^). Briefly, an overnight culture of *K. variicola* F2R9 lacking nalidixic acid was diluted 1:100 and then inoculated in fresh medium containing 16 μg ml^–1^ nalidixic acid. Thereafter, bacteria from the previous stage were serially passaged daily in Luria-Bertani broth (LB) containing increasing concentrations of nalidixic acid. Finally, the bacterium that grew in 128 μg ml^–1^ was considered a mutant, and this mutant was used as a recipient for conjugation experiments. The donor and recipient strains were cultured to logarithmic phase [optical density (OD) of approximately 0.6] at 37°C with shaking. Next, 200 μl of donor cells and 800 μl of recipient cells were mixed, placed into a new tube, and then incubated at 33°C overnight. After incubation, the mixture was serially diluted. Potential transconjugants were selected on LB agar plates containing 100 μg ml^–1^ rifampicin and 16 μg ml^–1^ potassium tellurite K_2_TeO_3_ for *E. coli* J53-2 transconjugants, and for *K. variicola* F2R9 transconjugants, plates contained 128 μg ml^–1^ nalidixic acid and 16 μg ml^–1^ potassium tellurite K_2_TeO_3_. The transconjugants were confirmed to have indistinguishable enterobacterial repetitive intergenic consensus (ERIC) patterns to the parent strains (Versalovic et al., 1991). Additionally, for *K*. *variicola* transconjugant analysis, we selected three genes of the *K. variicola* multilocus sequence typing scheme (Barrios-Camacho et al., 2019) that had a different allele compared with to *K. variicola* 8917. The *leuS*, *pgi*, and *pyrG* genes were selected for PCR amplification and Sanger sequencing (see Supplementary Table 2). Later, a phylogenetic analysis of the concatenated sequence of these three genes was performed. In addition, to confirm the acquisition of the plasmid, we amplified the plasmid markers *terW*, *fruA*, and *scrK* by PCR, and plasmid profile determination was performed according to the alkaline lysis method described by Kieser (1984). The *E. coli* NCTC 50192 strain, which contains 154-, 66-, 48-, and 7-kb plasmids, was used as a molecular size marker (Silva-Sanchez et al., 2013). The conjugation efficiency was calculated as the number of transconjugants per colony-forming unit (CFU) of recipients. *E. coli* J53-2 transconjugants were confirmed for auxotrophy to proline and methionine and for the presence of plasmid genes. Plasmid stability was determined by ten serial passages without potassium tellurite and the amplification of plasmid markers and plasmid extraction using alkaline lysis (Kieser, 1984).

### Plasmid Curing

Plasmid curing was performed according to El-Mansi et al. (2000). A solution of sodium dodecyl sulfate (SDS; 10% w/v, pH 7.4) was added to 2 × LB medium to give final concentrations of 0.5, 1, 2, 3, 4, and 5% SDS. An inoculum of 100 μl of a preculture of the tested strain was added to the SDS-containing LB and incubated at 27°C with shaking. Separated colonies were obtained by plating 100 μl of the cells in SDS-containing LB. Later, separated colonies were replicated on LB agar and LB agar containing potassium tellurite (16 μg ml^–1^). Colonies that were unable to grow in LB supplemented with potassium tellurite were considered as possible plasmid-free cells (cured strain). Plasmid-free cells were confirmed by analyzing the plasmid profile, amplification of the *terW, fruA*, and *scrK* genes, and ERIC-PCR to confirm the genetic relationship of the cured strain and the parent strain. Plasmid-free cells were grown in MacConkey agar to evaluate the hypermucoviscous phenotype by string test and mucoviscosity assay.

### Mucoviscosity Assay

In addition to the qualitative string test, the sedimentation degree was assessed according to Bachman et al. (2015). An overnight culture was pelleted by centrifugation at 8,000 × *g* and resuspended in phosphate-buffered saline (PBS) to an OD600 of ∼1. The suspensions were centrifuged for 5 min at 1,000 × *g*, and the OD600 of the supernatants was measured. Final readings were normalized to the OD600 of the wild-type culture before centrifugation. The results are presented as the mean and standard deviation of the data of three experiments. *K. variicola* F2R9 and *E. coli* J53-2 were used as a negative control for comparing *K. variicola* 8917, *K. variicola* F2R9, and *E. coli* J53-2 transconjugants, and as a positive control, the strain *K. pneumoniae* 10271 *rmpA*^+^/KL2 (Catalan-Najera et al., 2019) was used.

### Extraction and Quantification of Capsule

Glucuronic acid (GA) content was extracted and quantified using a colorimetric assay, as previously described (Lin et al., 2009). Briefly, 500 μl of bacterial cultures was mixed with 100 μl of 1% zwittergent in 100-mM citric acid. Then, the mixtures were incubated at 50°C for 20 min and centrifuged 5 min at 8,000 × *g*; the capsular polysaccharide (CPS) was precipitated by adding 1 ml of absolute ethanol to 250 μl of supernatants. Pellets were dissolved in 200-μl water, and then, 1,200 μl of 12.5-mM borax concentrated in H2SO4 was added. Mixtures were vigorously vortexed, boiled for 5 min, and cooled. Twenty microliters of 0.15% 3-hydroxydiphenol in 0.5% NaOH solution was added to the mixture, and the absorbance was measured at 520 nm. The GA content was determined from a standard curve of GA and expressed in microgram/10^9^ CFU.

### Phagocytosis Resistance Assay

The phagocytosis assay was performed as described elsewhere (Ares et al., 2016). In brief, THP-1 (ATCC TIB-202) human monocytes (differentiated to macrophages with 200-nM phorbol 12-myristate 13-acetate for 24 h; 6 × 10^5^) were seeded into 24-well tissue culture plates. Bacteria were grown in 5 ml of LB to the exponential phase. Macrophages were infected at a multiplicity of infection of 100 in a final volume of 1-ml Roswell Park Memorial Institute 1640 tissue culture medium supplemented with 10% heat-inactivated fetal bovine serum. To synchronize the infection, plates were centrifuged at 2,000 × *g* for 5 min. Plates were incubated at 37°C under a humidified 5% CO^2^ atmosphere. After 2 h, cells were rinsed three times with PBS and incubated for an additional 60 min with 1 ml of Roswell Park Memorial Institute 1640 containing 10% fetal bovine serum and gentamicin (100 μg ml^–1^) to eliminate extracellular bacteria. Cells were then rinsed again three times with PBS and lysed with 0.1% Triton X-100. After homogenization, 10-fold serial dilutions were plated onto LB agar plates to determine total CFUs.

### Serum Killing Assay

Human blood was obtained from healthy individuals, and resistance to serum killing was performed as previously described (Hughes et al., 1982; Podschun et al., 1991). An inoculum of 25 μl of bacterial suspension (∼10^6^ CFU) prepared from the mid-log phase was mixed with 75 μl of pooled human serum. Viable counts were checked at 0, 1, 2, and 3 h of incubation at 37°C. Each strain was tested three times, and the mean results were expressed as a percent of surviving inoculum. The response to serum killing in terms of viable counts was scored using six grades classified as serum sensitive (grade 1 or 2), intermediately sensitive (grade 3 or 4), or serum resistant (grade 5 or 6). Inactivated serum at 56°C was used as control.

### Mouse Infection Model

We implemented healthy and diabetic models. Healthy male BALB/c mice were obtained from the animal facility of the National Institute of Public Health at the age of 6–7 weeks old. Diabetic mice were induced through two doses of intraperitoneal injection of the β-pancreatic cell toxin streptozotocin; the first dose was 75 mg/kg, and the second dose was 135 mg/kg. Last, glucose was measured after 6 days of post-induction, and we considered diabetic conditions > 200 mgdl^−1^ glucose. *K*. *variicola* 8917, F2R9, and ΔpKV8917 were inoculated into healthy and diabetic mice as follows. Bacterial cell cultures were centrifuged at 10,000 × *g* for 5 min. The supernatants were discarded and the bacterial pellets resuspended in 1 ml of PBS solution. The solution containing PBS and bacterial cells was diluted, and 100 μl containing 1 × 10^8^ to 8 × 10^8^ was injected via the intraperitoneal route in healthy and diabetic mice. Animals were monitored twice daily for 10 days of post-inoculation. The transconjugant F2R9_TC14 was inoculated as described earlier in healthy mice. Animals were monitored twice daily for 10 days of post-inoculation, after which mice were killed. All animal studies were approved by the Biosafety Committee at the National Institute of Public Health.

### Whole-Genome Sequencing of *Klebsiella variicola* 8917

The sequence of the complete nucleotide sequence from the chromosome and plasmid of *K. variicola* 8917 was determined using long (MinION) and short-read (Illumina MiSeq) sequencing. For MiSeq sequencing, genomic DNA was extracted using the DNeasy Blood and Tissue Kit (QIAGEN Inc., Germany). For MinION sequencing, genomic DNA was extracted using the blood and cell culture kit. DNA concentrations were measured using the Qubit dsDNA HS Assay Kit (Fisher Scientific Inc.) on a Qubit 3.0 fluorometer (Fisher Scientific Inc.). *De novo* hybrid assembly of the short Illumina reads and long MinION reads was performed using Canu v2.0 and SPAdes v3.1.1 assemblers (Bankevich et al., 2012; Koren et al., 2017). Chromosome and plasmid were annotated under National Center for Biotechnology Information’s Prokaryotic Genome Annotation Pipeline. The sequence type of the *K. variicola* genomes included in this study was determined according to the *K. variicola* multilocus sequence typing platform^1^. The identification of virulence genes and plasmid replicon type was determined by the BLASTn search and PlasmidFinder 2.1 tools found in the Center for Genomic Epidemiology^2^.

### Comparative Genome and Plasmid Analysis

Plasmid and genome comparisons with other structures were generated by BLAST Ring Image Generator v.0.95.22 (Alikhan et al., 2011) using 90 and 70% as the upper and minimum thresholds, respectively. We conducted a genome comparison between three *K. variicola* genomes: 8917, TUM111415 (accession no. BIKO01000005.1), and KvL18 (accession no. PRJNA612181). The plasmid comparison was carried out by aligning the plasmids pKV8917, p15WZ-82_Vir (accession no. CP032356.1), pVir_030666 (accession no. CP027063.3), and pKSB1_10J unnamed2 (accession no. CP024517.1) and the chromosomes of *Raoultella terrigena* strain NCTC13098 (accession no. NZ_LR131271.1), *Pantoea coffeiphila* (accession no. NZ_PDET01000010.1), and *Kosakonia radicincitans* strain DSM 16656 (accession no. CP018016.1). The complete sequences of the chromosome and plasmid of *K. variicola* 8917 were updated in the GenBank database (CP063403 and CP063404).

### Identification of Capsule Mutations

Capsular types were determined from genome sequence data based on a complete K-locus sequence using the *Klebsiella* K locus primary reference database of the Kaptive v.0.5.1 (Wyres et al., 2016). The nucleotide sequence of the capsule operon of *K. variicola* 8917 was used as a query in BLASTn to look for strains with the same capsule type. *K. pneumoniae* strain QMP (accession no. LT174583.1), *K. variicola* 13450 (accession no. CP026013.1), and *E. coli* strain CSF3273 (accession no. CP026932.2) were identified as capsule-type KL114. Each protein sequence in the *cps* (comprising GalF and Ugd) was used to align with CLUSTALW to look for mutations in the capsule biosynthesis genes of the *K. variicola* 8917 genome. A phylogenetic tree was constructed with the concatenated amino acid sequence of the capsule cluster using MEGAX software. Easyfig (Sullivan et al., 2011) was used to compare the capsule genes.

### Statistical Analysis

For significant differences, we implemented an unpaired two-sided Student’s *t*-test and one-way analysis of variance. For survival curves, two-tailed Mann–Whitney *U*-tests were performed using Prism 8.0 software (GraphPad Software Inc., San Diego, CA, United States).

## Results

### Molecular and Genetic Characteristics of the *Klebsiella variicola* 8917 Isolate

*Klebsiella variicola* 8917 clinical isolate was previously identified by the string test, and a viscous filament greater than 5 cm long was observed; thus, it was considered hypermucoviscous (Garza-Ramos et al., 2015). Subsequently, a ∼200-kb plasmid designated pKV8917 was determined by the Kaiser method (Rodriguez-Medina et al., 2019). Nonetheless, plasmid assembly showed a larger plasmid of 343 kb that did not carry any known antimicrobial resistance genes and encoded tellurium resistance (Rodriguez-Medina et al., 2019). Core virulence factors were predicted from the genome sequence, including the iron transport system Kfu (*kfuABC*), the siderophore enterobactin (*entB*), and the type 3 fimbriae (*mrkABCDFHIJ*). This strain encoded neither colibactin, aerobactin, nor yersiniabactin. However, we found the receptor of aerobactin (*iutA*; Martinez-Romero et al., 2018; Rodriguez-Medina et al., 2019). Further analysis of the implications of carrying siderophore receptors for iron scavenging is required.

### pKV8917 Plasmid Confers Hypermucoviscosity-Like in *Klebsiella variicola* but Not in *Escherichia coli*

Mating experiments using *K. variicola* F2R9 and *E. coli* J53-2 as recipient strains showed that the hmv-like could be transferred to a non-hmv *K. variicola* but not to *E. coli* (Table 1). The plasmid markers *terW*, *scrK*, and *fruA* were amplified in the transconjugant, and resistance to tellurite was observed, confirming that the transconjugants acquired the plasmid (Table 1). In addition, the ERIC profile and the concatenated sequence of the *leuS*, *pgi*, and *pyrG* genes of F2R9 transconjugants were identical to the F2R9 parent strain (Supplementary Figures 1, 2). Likewise, the ERIC profiles of the *E. coli* transconjugants were identical to those of the recipient strain J53-2 (Supplementary Figure 1).

**TABLE 1:** Phenotypic and molecular characteristics of *K. variicola* and transconjugants.

Bacterial	Strains	Transconjugant	KL^*a*^	Plasmid	String	Mucoviscosity	Wild type			Acquired			Plasmid			Serum	Conjugation	Statistical
Species				profile (kb)^*b*^	Test^*c*^	assay^*d*^	phenotype			phenotype			markers^*e*^			resistance^*g*^	frequency	analysis^*d*^
										
							Rif	Tel	NA	Rif	Tel	NA	*terW*	*scrK*^*f*^	*fruA*^*f*^			
*K. variicola*	8917		114	∼300	+	0.24	S	R	S	NA	NA	NA	+	+	+	IS		*P* = 0.005
	ΔpKV8917		114	Negative	–	0.15	S	S	S	S	S	S	–	–	–	IS		*P* = 0.005
	F2R9		16	Negative	–	0.14	S	S	R	NA	NA	NA	–	–	–	S		
		F2R9_TC5	16	∼300	+	0.22	NA	NA	NA	S	R	R	+	+	+	S	1.4 × 10^–8^	*P* < 0.0001
		F2R9_TC14	16	∼300	+	0.21	NA	NA	NA	S	R	R	+	+	+	S	1.4 × 10^–8^	*P* < 0.0001
*E. coli*	J53-2		ND	Negative	–	0.05	R	S	R	NA	NA	NA	–	–	–	S		
		J53-2_TC5	ND	∼300	–	0.05	NA	NA	NA	R	R	R	+	+	+	S	6.9 × 10^–2^	
		J53-2_TC7	ND	∼300	–	0.05	NA	NA	NA	R	R	R	+	+	+	S	6.9 × 10^–2^	

*^*a*^K locus was determined using Kaptive tool (Wyres et al., 2016). ^*b*^The plasmid profile was determined by Kaiser protocol, the size of the plasmid was determined by whole plasmid sequencing (see section “Material and Methods”). ^*c*^The hypermucoviscosity was determined by string test. ^*d*^Sedimentation assay was determined according to Bachman et al. (2015), and statistical analysis were determined. ^*e*^Plasmid markers were determined by PCR (see section “Material and Methods”). ^*f*^Gene products; *scrK*, Fructokinase; *fruA*, PTS-system, IIC component. ^*g*^Serum resistance determination; S, sensitive; IS, intermediately sensitive.Nomenclature and abbreviations: -, Negative; +, Positive; NA, not applicable; ND, not determined; S, susceptible; R, resistant; Rif, Rifampicin; Tel, Tellurium; and NA, Nalidixic Acid.*

The conjugation frequency was low (10^–8^ CFU) in *K*. *variicola* F2R9; in contrast, when using *E. coli* J53-2, the conjugation frequency was 10^–2^ CFU (Table 1). Plasmid stability was tested in the transconjugants by the amplification of plasmid markers and plasmid extraction using alkaline lysis. pKV8917 was maintained after 10 serial passages even without potassium tellurite (data not shown).

Interestingly, the *K. variicola* F2R9 transconjugants were string test positive; meanwhile, the *E. coli* J53-2 transconjugants showed a negative string test result (Table 1). However, the string displayed by the F2R9_TC5 and F2R9_TC14 transconjugants was not the same size as that displayed by the *K. variicola* 8917 strain. Thus, hmv-like is conferred by the pKV8917 plasmid. Considering the high frequency observed with *E. coli* J53-2, a second mating was assayed using J53-2_TC7 and J53-2_TC5 as donors of pKV8917 with *K. variicola* F2R9 as the recipient; however, this mating was unsuccessful.

### pKV8917 Plasmid Curing Resulted in the Loss of Hypermucoviscosity-Like

**FIGURE 1:**
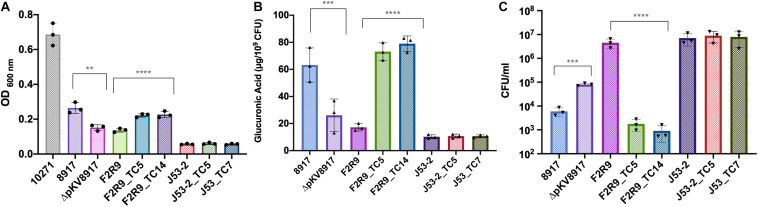
**(A)** Mucoviscosity, **(B)** glucuronic acid production, and **(C)** phagocytosis killing assays for *K. variicola* 8917, ΔpKV8971, F2R9, *E*. *coli* J53-2, and transconjugants. Each symbol represents the number of biologically independent experiments. Data are presented as the mean ± standard deviation of three independent experiments. ****P < 0.0001; ***P < 0.001; and **P = 0.005. Strain 10271 corresponds to a hypervirulent/hypermucoviscous *K. pneumoniae rmpA*^+^/KL2^+^ (Catalan-Najera et al., 2019) and was used as a reference for non-sedimentation behavior.

Bacterial cells recovered with 5% SDS were selected for further analysis. One colony (ΔpKV8917 strain) was successfully cured from a total of 1,000 colonies analyzed. This was evidenced by (i) the loss of tellurite resistance, (ii) the absence of plasmid markers *terW, scrK*, and *fruA* (Table 1), and (iii) the absence of plasmid pKV8917 determined by the Kieser protocol (Supplementary Figure 3). Interestingly, growth on MacConkey agar showed the inability of the ΔpKV8917 strain to display the viscous filament, and mucoviscosity assay reflects a slight reduction in OD measurement (Figure 1A). The loss of hmv-like was observed at a frequency of 0.1%. This result confirms the relationship that exists between hmv and the pKV8917 plasmid.

### *Klebsiella variicola* 8917 Is Hypermucoviscous but Sediments at Low-Speed Centrifugation

The mucoviscosity assay has been recommended as a quantitative indicator of hmv. The tested strain is subjected to low centrifugation, and the supernatant is measured at OD 600. Usually, hmv strains have poor sedimentation (Walker and Miller, 2020). Although *K. variicola* 8917 is hypermucoviscous, it sedimented well at low-speed centrifugation (Figure 1A). When comparing the cured (ΔpKV8917) and transconjugant (F2R9_TC5 and F2R9_TC14) strains with their respective parent strain, a slight difference was observed (Figure 1A and Table 1). For a comparative purpose, we included an hvKpn-*rmpA* positive strain (*K. pneumoniae* 10271), and, as expected, it did not sediment well, showing a turbid supernatant (OD_600_ 0.6; Figure 1A).

### pKV8917 Plasmid Acquisition Increases Capsule Production and Impacts Phagocytosis by THP-1 Macrophages but Not Serum Resistance

Glucuronic acid (GA) is a type of uronic acid precursor sugar nucleotide and a common component of capsules and O antigens (Shu et al., 2009). GA was measured to determine the amount of CPS produced in the parent, cured, and transconjugant strains. Figure 1B indicates that *K. variicola* 8917 produced 63.2 μg of GA μg/10^9^ CFU, whereas ΔpKV8917 showed a significant reduction in the production of GA of more than 50% (*P* < 0.001); however, the elimination of the plasmid did not completely abolish the capsule. Growth on MacConkey agar indicated that ΔpKV8917 and 8917 strains maintained the mucoid colony phenotype typically associated with the presence of a capsule.

Phagocytosis is a crucial process to eliminate pathogens at the early stages of infection; however, many bacteria have developed strategies to avoid phagocytosis and have become “resistant” to the action of neutrophils and macrophages, which are major components of the innate immune response (Medzhitov, 2007). Capsules are by far the main virulence factor that protects bacteria against the host immune response by inhibiting phagocytosis and lysis by complement and antimicrobial peptides (Paczosa and Mecsas, 2016). In this study, we compared the level of phagocytosis in the parent, cured, and transconjugant strains. Our results showed that *K. variicola* 8917 is less phagocytosed by THP-1 macrophages than the cured strain ΔpKV8917 (*P* < 0.001; Figure 1C). Additionally, the F2R9_TC5 and F2R9_TC14 transconjugants were also less phagocytosed by macrophages compared with the parent strain F2R9 (*P* < 0.0001; Figure 1C). These data correlate with the changes brought by the loss or acquisition of pKV8917 over the production of CPS. This effect was not observed in *E. coli* J53-2 or its transconjugants because despite containing the pKV8917 plasmid, these strains did not display hmv-like properties (Figure 1A and Table 1). Serum resistance assay showed grade 2 (sensitive) for *K. variicola* F2R9, F2R9_TC5, F2R9_TC14, *E. coli* J53-2, J53-2_TC5, and J53-2_TC7 and grade 3 (intermediately sensitive) for *K. variicola* 8917 and *K. variicola* Δp8917 according to the classification of Podschun R et al. (Table 1).

### Loss or Acquisition of the pKV8917 Plasmid Drives Differences in Virulence

**FIGURE 2:**
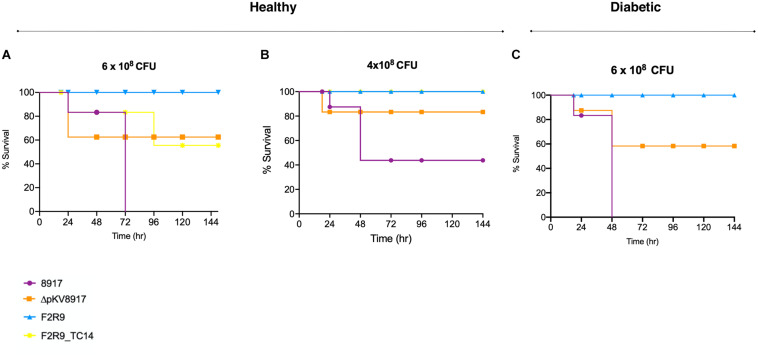
Survival curves of *K. variicola* 8917, ΔpKV8917, and F2R9_TC14 in a healthy mouse model using two different CFUs **(A,B)**. Diabetic mice were infected with 6 × 10^8^CFU **(C)** of *K. variicola* 8917 and ΔpKV8917. Non-hmv strain *K. variicola* F2R9 was inoculated at the higher dose tested (8 × 10^8^) and did not cause death. Significance was calculated with the Mann–Whitney two-tailed *U*-test.

The level of virulence was evaluated in healthy and diabetic mice in an induced sepsis model. The mortality rate of *K. variicola* 8917 at 6 × 10^8^ CFU was 100% after 72 h; this effect was not observed for the ΔpKV8917 strain at the same bacterial dose (Figure 2A). Otherwise, mice were infected with 4 × 10^8^ CFU *K. variicola* 8917 and ΔpKV8917, 40 and 80% survival rates were observed, respectively (Figure 2B). Clearly, the virulence of the ΔpKV8917 strain decreased, although the mortality rate was 40% at the highest dose inoculated. Note that lower doses of the ΔpKV8917 strain did not result in mortality; moreover, the inoculation of *K. variicola* F2R9, which was used as a non-hmv strain, did not result in mortality, even at high doses.

Most importantly, mice that were infected with 6 × 10^8^ of F2R9_TC14 transconjugant showed 40% mortality at 96 h (Figure 2A); however, mortality of 0% was observed at a lower dose (4 × 10^8^ CFU; Figure 2B). Thus, the level of virulence of the F2R9_TC14 transconjugants was higher than that of F2R9.

Diabetes is considered a risk factor for infections caused by *K. pneumoniae*, especially for hypervirulent strains (Catalan-Najera et al., 2019); however, the risk factor for hmv-like strains has not been assessed. Using a diabetic mouse model, we evaluated whether this condition could affect the survival rate when mice are challenged with an hmv-like strain compared with a non-hmv strain. Diabetic mice inoculated with 6 × 10^8^ CFU of *K. variicola* 8917 showed 100% mortality at 48 h in contrast to healthy mice (72 h; Figure 2C). A fast killing was not observed with *Δ*pKV8917 and *K. variicola* F2R9 in diabetic mice at the same bacterial dose inoculated (Figures 2A,C).

### pKV8917 Has an IncFIB Backbone but a Unique Genetic Makeup

**FIGURE 3:**
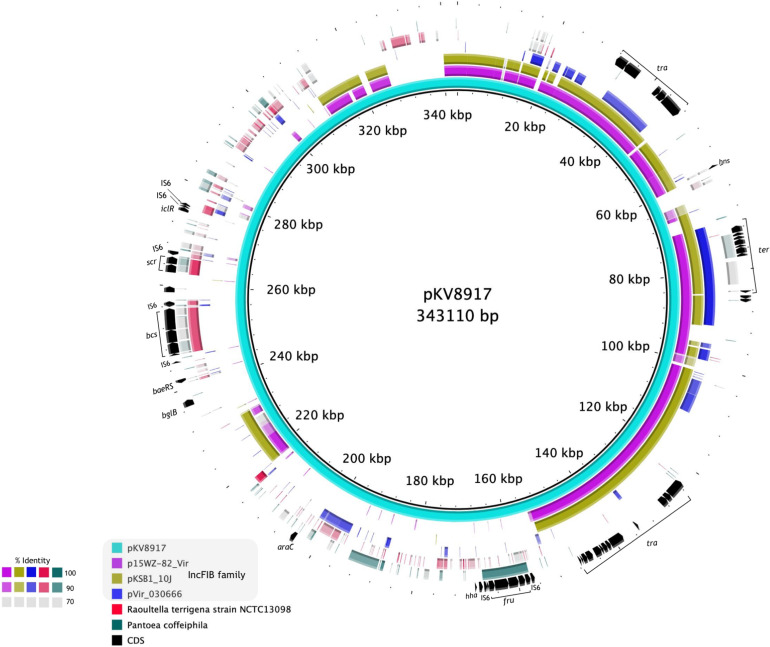
Alignment of the complete sequence of pKV8917 with two plasmids of hypervirulent *K*. *variicola* strains (p15WZ-82_Vir and pVir_030666) and one plasmid of a multidrug-resistant CTX-M-15-producing *K. pneumoniae* strain (pKSB1_10J). We also included the chromosomes of *R. terrigena* NCTC13098 and *P. coffeiphila.%* of nucleotide identity for each region aligned is indicated in the color legend.

A diagram of pKV8917 (343-kb) is shown in Figure 3. The complete sequence of the pKV8917 plasmid was compared with p15WZ-82_Vir (282,290-bp; Yang et al., 2019), pVIR_030666 (235,448-bp; Lu et al., 2018), and unnamed2 plasmid (212,079-bp) recovered from a CTX-M-15-producing *K. pneumoniae* strain KSB1_10J (Gorrie et al., 2018). pKV8917 exhibited 99% identity with 49% coverage to the virulence plasmid p15WZ-82_Vir and pKSB1_10J unnamed2 plasmid, both self-transmissible (Figure 3). The common regions encoded conjugation machinery (*tra*), tellurium resistance cluster (*ter*), histone-like nucleoid protein (H-NS), plasmid maintenance genes (*parAB*/*sopAB*), type I toxin-antitoxin (TA) system (*hokB*), and error DNA repair system (*umuCD*) and several hypothetical proteins (Figure 3). Another common feature was the incompatibility group IncFIB; however, pVir_030666 contained an additional replicon family, IncHI1B (Lu et al., 2018).

Further sequence analysis showed the identification of regions aligned with the chromosomes of *P. coffeiphila* and *R. terrigena* NCTC13098 (Figure 3). We identified a cellulose biosynthesis cluster (*bcs*) and a fructose-dependent (*fru*) phosphotransferase system (PTS) and two genes encoding a sucrose-dependent (*scr*) PTS. The *bcs* cluster was also harbored by *R. terrigena* NCTC13098 and five genomes of *K. variicola* (VRCO0297, VRCO0299, VRCO0486, VRCO0296, and VRCO0300 from the BioProject PRJEB18814). The fru*-*PTS system had a significant identity to a PTS from *Pantoea coffeiphila.* Genes encoding the fru-PTS and the cellulose biosynthesis cluster were flanked by two insertion sequences (IS6), suggesting its mobilization and integration into the pKV8917 plasmid (Figure 3). The two genes encoding a sucrose PTS (*scr*) had a significant identity with genes from *R. terrigena* NCTC13098. The transcriptional regulators *araC* and *iclR* were also found in pKV8917 (Figure 3); they exhibited high similarity and coverage to *araC* and *iclR* regulators found in *Kosakonia* species. Although pKV8917 shares regions with known plasmids, its genetic structure is unique.

Moreover, we identified two clinical isolates reported as hmv-like Kv18L and TUM14115 obtained from endodontic infection and bacteremia, respectively (Harada et al., 2019; Nakamura-Silva et al., 2020). A BLAST search of the *rmpADC* and *rmpA2* genes against these two genomes confirmed their absence. Genome alignment between 8917, Kv18L, and TUM14115 showed a ∼95% nucleotide identity (Supplementary Figure 4). BLAST analysis revealed that TUM111415 lacked a plasmid replicon because all contigs corresponded to chromosomal hits, whereas Kv18L showed several plasmid-associated contigs. As there were no plasmid assembly data for Kv18L and TUM111415 that apparently lack plasmid replicon, we were not able to perform a plasmid comparison between pKV8917 and other plasmids of *K. variicola* strains with hmv-like properties.

### Analysis of Capsule Locus of hmv-Like *Klebsiella variicola*

Analysis of mutations in the capsule protein-coding genes showed that although the entire K-Locus is conserved among KL114 capsule-type strains, there are several amino acid substitutions across the CPS proteins (Supplementary Figure 5 and Supplementary Table 3). The glycosyltransferase (GT_KL114) was the most diverse gene with 16 aminoacid substitutions. In addition, we observed that the capsule loci of both *K. variicola* strains are more closely related compared with *K. pneumoniae* (Supplementary Figure 5). Because we do not know the phenotypic traits of the *K. variicola* 13450 and *K. pneumoniae* QMP strains, we are not able to associate the mutations in capsule biosynthesis genes of *K. variicola* 8917 with hmv, and so, this need to be further investigated.

Diverse capsule types were identified in other hmv-like *K. variicola*, Kv18L and TUM14115 isolates, which were KL34 and KL125 capsule types, respectively. Additionally, *manCB* genes, which are responsible for GDP-D-mannose synthesis, a common component of capsules, were identified in F2R9 and 8917 capsule loci. On the other hand, the hypervirulent *K. variicola* isolates used for plasmid comparison (15WZ-82 and WCHKP030666) both possessed the KL16 capsule type.

## Discussion

*Klebsiella variicola* 8917 showed a hypermucoviscous-like phenotype; however, it does form a pellet upon centrifugation as non-hmv strains. *K. variicola* 8917 seems to behave as non-hmv when compared with *K. pneumoniae* 10271, a hv-hmv and KL2 strain (Catalan-Najera et al., 2019). We suggest that the sedimentation property in *K. variicola* 8917 depends on its polysaccharide, lipid composition, or hmv composition, which could vary between these hmv strains and may lead to non-sedimentation typically associated with hv-hmv *K. pneumoniae*.

When the pKV8917 plasmid was acquired by *K. variicola* F2R9, we noted that its transconjugants (F2R9_TC5/TC14) produced more capsule than *K. variicola* 8917 and F2R9 (Figure 1B). Notably, the J53-2_TC7 and J53-2_TC5 transconjugants maintained the same production level of CPS production, consistent with the results of mucoviscosity assay and the absence of hmv-like (Figures 1A,B). The F2R9_TC5 and F2R9_TC14 transconjugants were also less phagocytosed by macrophages compared with their parent strain F2R9 (*P* < 0.0001). However, the serum resistance assay showed a serum sensitive grade, and so it seems that hmv-like is advantageous to evade phagocytosis but do not prevent the bactericidal action of the complement system (Table 1). Phagocytosis and production of CPS correlate with the changes brought about by the loss or acquisition of pKV8917, but inconsistently, the measurement of mucoviscosity was lower than expected; although the string test was positive, the filament in transconjugants was not the same size as that produced by *K. variicola* 8917. A similar result was also observed in the work by Yang et al. (2019), who transferred the p15WZ-82_Vir virulence plasmid to several *Klebsiella* strains. Further analysis of the effects of acquiring an hmv-like-plasmid by strains with the same capsule type may explain why hmv was not exhibited at the same level as the plasmid-harboring strain.

To date, the number of reports worldwide of hmv-like *K. variicola* is limited (Garza-Ramos et al., 2015; Harada et al., 2019; Imai et al., 2019; Nakamura-Silva et al., 2020), and particularly in Mexico, there are no additional reports. The misclassification of *K. variicola* as *K. pneumoniae* may impact the identification of *K. variicola* hmv-like strains.

Altogether, mating experiments and plasmid curing suggest the presence of a novel mechanism involved in both hmv and hyperproduction of the capsule in *K. variicola* 8917 in an unknown interacting network. Furthermore, we assessed the impact of the acquisition of the pKV8917 plasmid in a non-hmv isolate and its elimination in *K. variicola* 8917 on virulence level and phagocytosis. The F2R9_TC14 and F2R9_TC5 transconjugants were less phagocytosed than the F2R9 strain. This effect is likely associated with the presence of hmv-like and hyperproduction of the capsule, which have been related to high resistance to phagocytic uptake by macrophages (Paczosa and Mecsas, 2016). Although *K. variicola* F2R9 possesses “avirulent” behavior, F2R9_TC14 had a mortality of 40%; thus, the acquisition of pKV8917 led to an increase in virulence. In contrast, the ΔpKV8917 strain was phagocytosed in greater numbers than *K. variicola* 8917, which is consistent with a reduction in capsule, hmv production, and virulence.

Other plasmid-encoded virulence determinants could also contribute to virulence, including the tellurite resistance cluster (*terZABCDE* and *terW*), which is highly distributed in hvKpn strains and other species of the *K. pneumoniae* complex with hmv phenotype (Arena et al., 2015; Garza-Ramos et al., 2016; Martinez-Romero et al., 2018). The tellurite resistance cluster has been considered as a defense system involved in the pathogenesis (Yang et al., 2019).

Likewise, our data showed that diabetic mice challenged with hmv-like *K. variicola* 8917 have worse outcomes, with rapid death of the total mouse population occurring in less time and are more prone to infection than mice infected with a non-hmv strain (F2R9).

*Klebsiella variicola* occupies environmental niches (Rodriguez-Medina et al., 2019); thus, pKV8917 may have originated from environmental strains such as *Pantoea* sp., *Raoultella* sp. (formerly *Klebsiella terrigena*), and *Kosakonia* sp. It has been previously suggested that *K. variicola* could be transmitted to humans by phytonosis (Martinez-Romero et al., 2018; Rodriguez-Medina et al., 2019). *K. variicola* shares some environmental niches with *Pantoea, Raoutella*, and *Kosakonia*, which may interchange genetic material. Seemingly, plasmid recombination events occurred among *K. variicola* and *K. pneumoniae* bacterial species resulting in a chimeric plasmid with some genetic regions belonging to *Pantoea, Raoutella*, and *Kosakonia* and incFIB self-transmissible plasmid from *K*. *pneumoniae* (Supplementary Figure 6). This scenario is plausible considering that the incompatibility group IncFIB identified in pKV8917 is highly disseminated among plasmids of clinical and community strains of *K. pneumoniae* (Wyres et al., 2020).

The RmpADC-pathway is a complex regulatory cascade that triggers hmv in hypervirulent *K. pneumoniae*. We suspect that hmv-like-associated genes in *K. variicola* 8917 may come from environmental strains and may be related to genes involved in metabolic fitness, particularly those implicated in sugar transport such as the fructose- and sucrose-PTS. The metabolic cost for assembling the capsule and the production of hmv could be high and requires more precursors for completing both processes; this may explain the role of the PTSs in hmv-like and capsule synthesis (Figure 3).

The biosynthesis of cellulose by *Klebsiella* has not been described. This feature is maintained throughout the plant kingdom and some bacterial species, such as *Rhizobium* sp. and *Acetobacter* sp. (Saxena et al., 1994). Intriguingly, we identified other isolates of *K*. *variicola* harboring the cellulose cluster. Cellulose is an extracellular complex polysaccharide; however, we do not know whether cellulose-loci in *K. variicol*a 8917 could add to the hmv-like. Finally, the involvement of the transcriptional regulators *araC* and *iclR* in regulating vast processes such as carbon metabolism, virulence, and the glyoxylate bypass is intriguing because they may be participating indirectly in the regulatory circuit of the hmv-like (Gallegos et al., 1997; Molina-Henares et al., 2006). Additional experiments are required to assess the role of these genes over the hmv-like in *K. variicola* 8917.

The increasing number of hmv-like *K. pneumoniae* isolates is intriguing and suggests that other pathway(s) are involved. Recently, Ernst et al. (2020) reported novel capsule phenotypes mediated by deletions or single chromosomal mutations of core capsule biosynthesis genes among multidrug-resistant *K. pneumoniae* ST258. They found hypercapsulated mutants due to a missense mutation in the *wzc* gene (G565S). This substitution was searched in the *K. variicola* 8917 isolate; however, no mutation in the *wzc* gene was found in that position, and we did not detect mutations in the *rcsAB* or *lon* genes (data not shown). Comparing the KL114 capsule-type of three different strains, we identified capsule locus variation throughout the *cps*. Noteworthy, the *cps* loci of both *K. variicola* were more similar than the KL114 of *K. pneumoniae* (Supplementary Figure 5). KL114 is a rare capsule type among *K. pneumoniae*; the presence of subtypes within KL114 may not have been addressed due to limited sequence data. Nonetheless, the composition and diversity of *K. variicola* capsule loci deserve to be studied in detail.

Hypermucoviscosity is detected more frequently, and multiple pathways can trigger it; the implications of this phenotype when a hypervirulent background is absent deserve further analysis because this phenotype may play a role as a virulence factor. In conclusion, the acquisition of pKV8917 plasmid confers the hmv-like phenotype triggering an increase in capsule and virulence, especially with risk in diabetic populations. This study proposes a novel plasmid-borne mechanism that could be disseminated among the *K. pneumoniae* complex.

## Data Availability Statement

The sequences of the chromosome and plasmid of *K. variicola* 8917 have been deposited in GenBank under accession numbers: CP063403 and CP063404, respectively. Bioproject PRJEB7831.

## Ethics Statement

The animal study was reviewed and approved by Biosafety Committee at National Institute of Public Health.

## Author Contributions

UG-R and NR-M: conceived and designed the experiments. NR-M, MD, MA, and HV-T: performed the experiments. UG-R, NR-M, and LL-A: provided analytical tools. UG-R, JS-S, VA, and JM-B: contributed reagents and materials. NR-M, EM-R, and UG-R: wrote the manuscript. All authors contributed to the article and approved the submitted version.

## Conflict of Interest

The authors declare that the research was conducted in the absence of any commercial or financial relationships that could be construed as a potential conflict of interest.

## Footnotes

1 http://mlstkv.insp.mx

2 https://cge.cbs.dtu.dk/services/PlasmidFinder/

## Supplementary Material

Supplementary Figure 1 ERIC profiles of the (a) 8917 and F2R9 parent strains and transconjugants F2R9_TC5/14 and J53-2_TC5/7 and (b) ΔpKV8917 cured strain. The red symbol represents a possible plasmid fragment of ∼500 bp.

Supplementary Figure 2 Phylogenetic tree based on the concatenated sequence of *pgi*, *leuS*, and *pyrG* genes. The phylogeny was generated with the clustering method neighbor joining using MEGAX software.

Supplementary Figure 3 Plasmid profile based on alkaline lysis. Lines 1 and 10, *E. coli* NCTC 50192 plasmids were used as molecular size markers (154-, 66-, 48-, and 7-kb; Bachman et al., 2015); lines 2-3, *K. variicola* 8917 (pKV8917); line 4, ΔpKV8917 (cured strain); lines 5-6, J53-2_TC5 and J53-2_TC7; lines 7-8, F2R9_TC5 and F2R9_TC14; and line 9, F2R9.

Supplementary Figure 4 Genome sequence alignment of 8917 vs TUM14115 and Kv18L obtained from hmv *K. variicola* isolates.

Supplementary Figure 5 Phylogeny of isolates with capsule-type KL114. The phylogenetic tree was based on the concatenated amino acid sequence of 19 proteins that make up KL114 capsular type. *K. variicola* strains 13450 and 8917 are indicted with a triangle, *K. pneumoniae* strain QMP with black square and *E. coli* strain CFS3273 with black circle is showed. In addition, the comparison of the complete KL114 capsule locus. CDSs are presented as arrows. Gray bars indicate regions of similarity, and darker regions indicate a higher degree of similarity. Red arrows correspond to mannose synthesis and processing proteins (ManC and ManB). The number of mutations found across the *cps* is represented with blue bars. Detailed information on the sites of mutation is in Supplementary Table 3.

Supplementary Figure 6 A model for how the pKV8917 may have originated. **(A)** *K*. *variicola* is ubiquitous in soil, plants, animals and water. These natural niches possess a diverse bacterial population and allow *K. variicola* to coexist with other endophytes and bacteria of animal carriage. *Pantoea* sp. *Kosakonia* sp. and *Raoultella* sp. were donors of genetics regions to *K*. *variicola* 8917 possibly by horizontal transfer. *Raoultella* sp. is isolated more frequently from animal gut and sewage so that *K. variicola* 8917 might have acquired the cellulose biosynthesis cluster (*bcs*) in this spot. Our hypothesis is that hypermucoviscosity was acquired by bacterial species from the environment. By phytonoses, humans become infected by *K. variicola* 8917 which could be transported to the community **(B)** or hospital setting **(C)** in this scenario *K*. *variicola* coexist with *K. pneumoniae.* IncFIB plasmids (IncFIBp) are usually associated with multidrug-resistant and virulent strains; IncFIBp carries the transference module (*tra*) and tellurium resistance operon (*ter*), IncFIBp was transferred by means of conjugation to hmv-like *K. variicola* 8917. IncFIBp serves as the backbone for the incorporation of the genetic elements acquired from environmental strains resulting in the structure pKV9817. pKV8917 could be transfer the hmv-like to species of the *K. pneumoniae* complex **(D)** but also acquire virulence and multidrug-resistance genes.
