# Supplementary material for: A Klebsiella variicola Plasmid Confers Hypermucoviscosity-Like Phenotype and Alters Capsule Production and Virulence
Source: Front Microbiol. 2020 Dec 16;11:579612. doi: 10.3389/fmicb.2020.579612 (PMC7772424; doi:10.3389/fmicb.2020.579612)
Supplement: Supplementary file 1 [file Table_1.docx]

**Supplementary Table 1**. Characteristics and PCR conditions for amplification of plasmid markers and genetic fingerprint using the amplification of Enterobacterial Repetitive Intergenic Consensus (ERIC) sequences.

| **Primers and sequence (5’-3’)** | **Annealing (°C)** | **Gene** | **Amplification product (bp)** | **Reference** |
| --- | --- | --- | --- | --- |
| terW-F (TACACCCTGGCTAACATACT)  terW-R (TTTTTCTACTGCCTCGCTTC) | 58 | *terW* | 367 | This study |
| 940012-F (TGGTTCACTGATGGTAGCCG)  940012-R (CGGGTTTTTCAGCGTGAAGG) | 60 | *fruA* | 763 | This study |
| 940016-F (ATGAATACCTCCGGCGAGGA)  940016-R (AACTGGTCGGTAAACACCCG) | 60 | *scrK* | 776 | This study |
| ERIC1 (CACTTAGGGGTCCTCGAATGTA)  ERIC2 (AAGTAAGTGACTGGGGTGAGCG) | 52 | NA | NA | (Versalovic et al., 1991). |
| *leuS*Kv-F (CGAACAGGTTATCGACGGCT)  *leuS*Kv-R (CAAAGGTGTCGGTTTCACGC) | 63 | l*euS* | 968 | (Barrios-Camacho et al., 2019) |
| *pgi*Kv-F(AAAGAGACCGATCTGGCAGG)  *pgi*Kv-R (ACCAGATACCGATCAGCGCC) | 60 | *pgi* | 760 | (Barrios-Camacho et al., 2019) |
| *pyrG*Kv*-*F (CCGATCGCTATGGTCGCTG)  *pyrG*Kv*-*R (CGGGACATCAGTTCCGGGT) | 60 | *pyrG* | 664 | (Barrios-Camacho et al., 2019) |

**Supplementary Table 2.** MLST allelic profile of *K. variicola* 8917 and F2R9 isolates.

| **Strain** | **Type** | **Allelic Profile** | | | | | | |
| --- | --- | --- | --- | --- | --- | --- | --- | --- |
|  |  | ***leuS^a^*** | ***pgi^a^*** | ***pgk*** | ***phoE*** | ***pyrG^a^*** | ***rpoB*** | ***fusA*** |
| 8917 | Parental | 1 | 2 | 2 | 1 | 2 | 1 | 2 |
| F2R9 | Parental | 4 | 7 | 5 | 1 | 10 | 1 | 4 |
| F2R9_TC5 | Transconjugant | **4** | **7** | ND | ND | **10** | ND | ND |
| F2R9_TC14 | Transconjugant | **4** | **7** | ND | ND | **10** | ND | ND |

The allelic profile was consulted in the *K. variicola* MLST database^1^.

^a^These genes were selected for PCR amplification, sequencing and analysis using Neighbor Joining clustering method see **Supplementary Figure S2.** The sequences of these genes were uploaded from MLST K. variicola database^1^ and consulted the corresponding allele (bold numbers).

___________________________

^1^<http://mlstkv.insp.mx>

**Supplementary Table 3**. Mutations found in the KL114 capsule biosynthesis protein-coding genes of *K. variicola*

| **Genome** | **Species** | ***cps* proteins of KL114 locus** | | | | | | | | | | | | | | | | | | |  |
| --- | --- | --- | --- | --- | --- | --- | --- | --- | --- | --- | --- | --- | --- | --- | --- | --- | --- | --- | --- | --- | --- |
|  |  | **GalF** | **CpsAB** | **Wzi** | **Wza** | **Wzb** | **Wzc** | **WbaP** | **Wzy** | **WciP** | **Wzx** | **WcqZ** | **WcuQ** | **GT** | **WbaZ** | **ACT** | **Gnd** | **ManC** | **ManB** | **Ugd** | |
| 8917 | *K. variicola* | I153V  E253Q  M296L | A96T  V144L  S150T  D151H  P152S  S177N | K102Q  F195L  V315I | - | - | K587N | I178T | S251T | - | K202Q  I383M  M438I  L442F | I132V | I56V  I107V  D217V  D248G  R357Q | T22A  D62E  I68T  N89K  R99H  D123E  V124I  N135S  K147E  T308V  V412I | - | N110H  M289T  S317 | I50K  A167E  K217Q  D383G | Y31F  Q131H  G287E  V223A | M129L | - | |
| 13450 | *K. variicola* | I153V  T287A  M296L | A96T  V144L  S150T  D151H  P152S  S154C  S177N | K102Q  E132D  F195L  V315I | - | - | - | I178T | S251T | E179G  N265K | K202Q  I383M  M438I  L442F | I132V  T179I | I56V  I107V  E210D  D217V  D248G  R357Q | T22A D62E  I68T  N89K  G91S  R99H  D123E  V124I  N135S  K147E  T308V  Q332H  V412I  N446K | I367M | F36L  N110H  S317 | I50K  A167E  K217L  D383G  Q395K | Y31F  G287E  V223A | M129L | - | |
| CSF3273 | *E. coli* | I153V  E253Q  M296L | A137V  V144L  S150T  D151H  P152S  S154N  S177N | I4N  A61S  K102Q  V132I  N146S  M197L  A230G  T244S  Q266E  V315I | S31T | **-** | T352A  R606C | I178T | S251T  I302M | N265K | - | - | I266V | L21I  D62E  N135D | - | E200K | I50K  D383G | Y31F  H101Y  P120S  V223A  S236N | M129L  N167K | Y91H | |

The capsular polysaccharide biosynthesis gene cluster type KL114 of the strain *K. pneumoniae* QMP (Accession No. LT174583.1) was used as reference for comparison with other capsule-type KL114 isolates. Unique mutations are highlighted in red, share mutations in blue and mutations found in all isolates in black. *K. pneumoniae* and *K. variicola* strains share the same *wzi* and *wzc* alleles, 932 and 454 respectively; however, *wzi* allele of *E. coli* CSF3273 correspond to 355, thus, we do not include this gene in the count of mutations (**Supplementary Figure 5)**. Nomenclature and abbreviations: -, absence of mutations; GT, glycosyltransferase KL114; ACT, acetyltransferase KL114.
