# Supplementary figures and images for: A Klebsiella variicola Plasmid Confers Hypermucoviscosity-Like Phenotype and Alters Capsule Production and Virulence
Source: Front Microbiol. 2020 Dec 16;11:579612. doi: 10.3389/fmicb.2020.579612 (PMC7772424; doi:10.3389/fmicb.2020.579612)

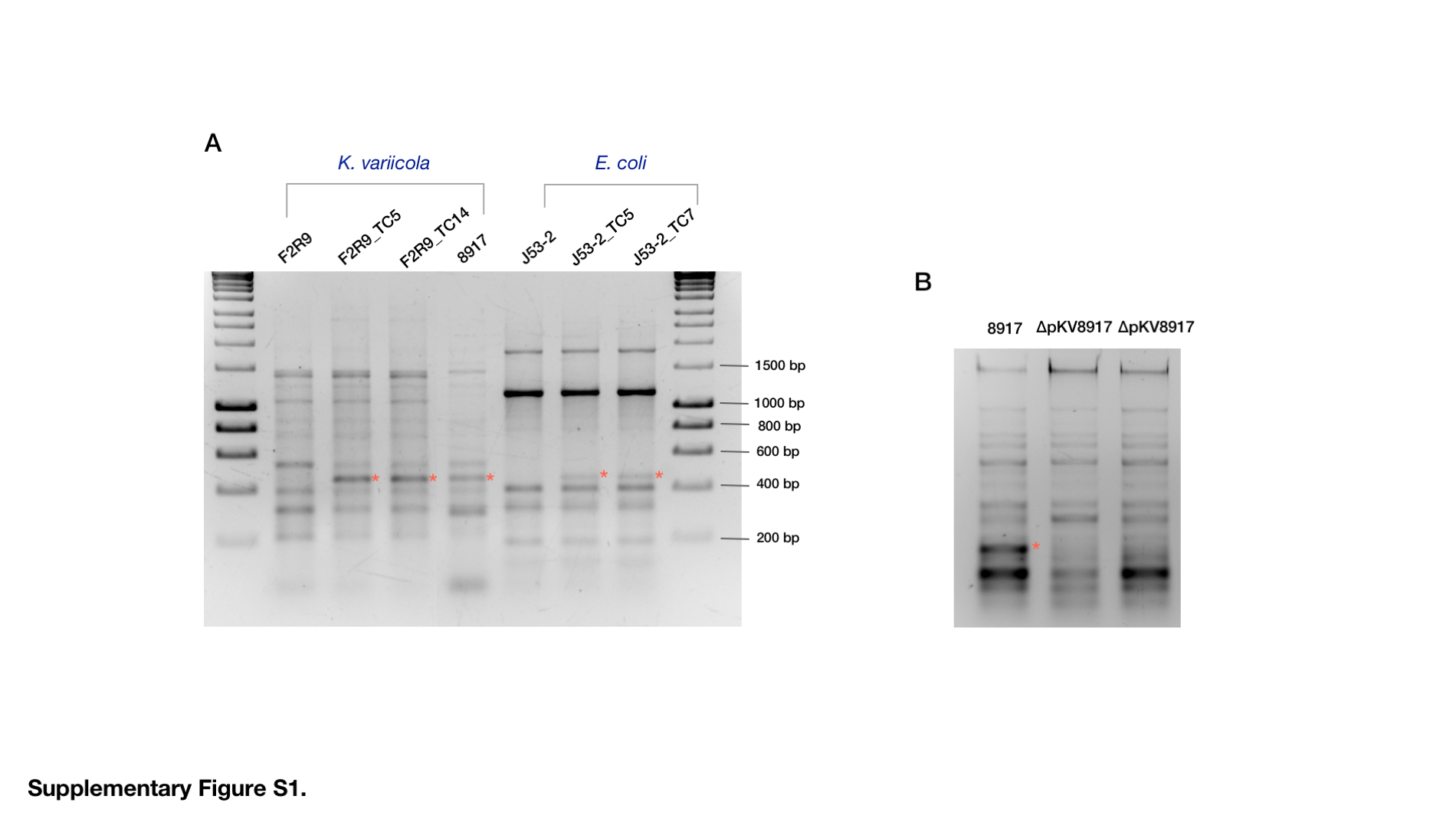

Supplement: Supplementary Figure 1 — ERIC profiles of the (a) 8917 and F2R9 parent strains and transconjugants F2R9_TC5/14 and J53-2_TC5/7 and (b) ΔpKV8917 cured strain. The red symbol represents a possible plasmid fragment of ∼500 bp. [file Image_1.tiff]

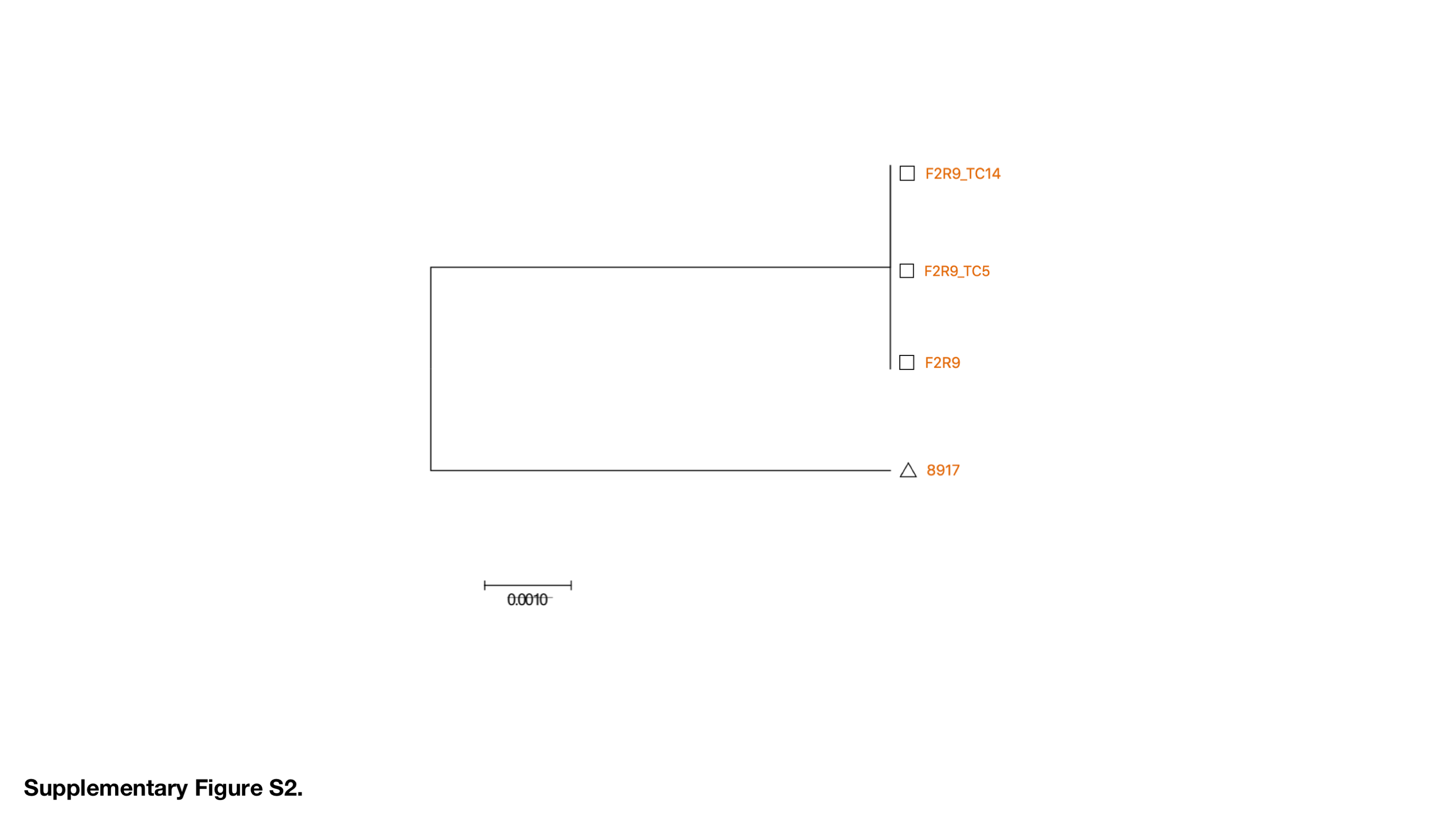

Supplement: Supplementary Figure 2 — Phylogenetic tree based on the concatenated sequence of pgi, leuS, and pyrG genes. The phylogeny was generated with the clustering method neighbor joining using MEGAX software. [file Image_2.TIFF]

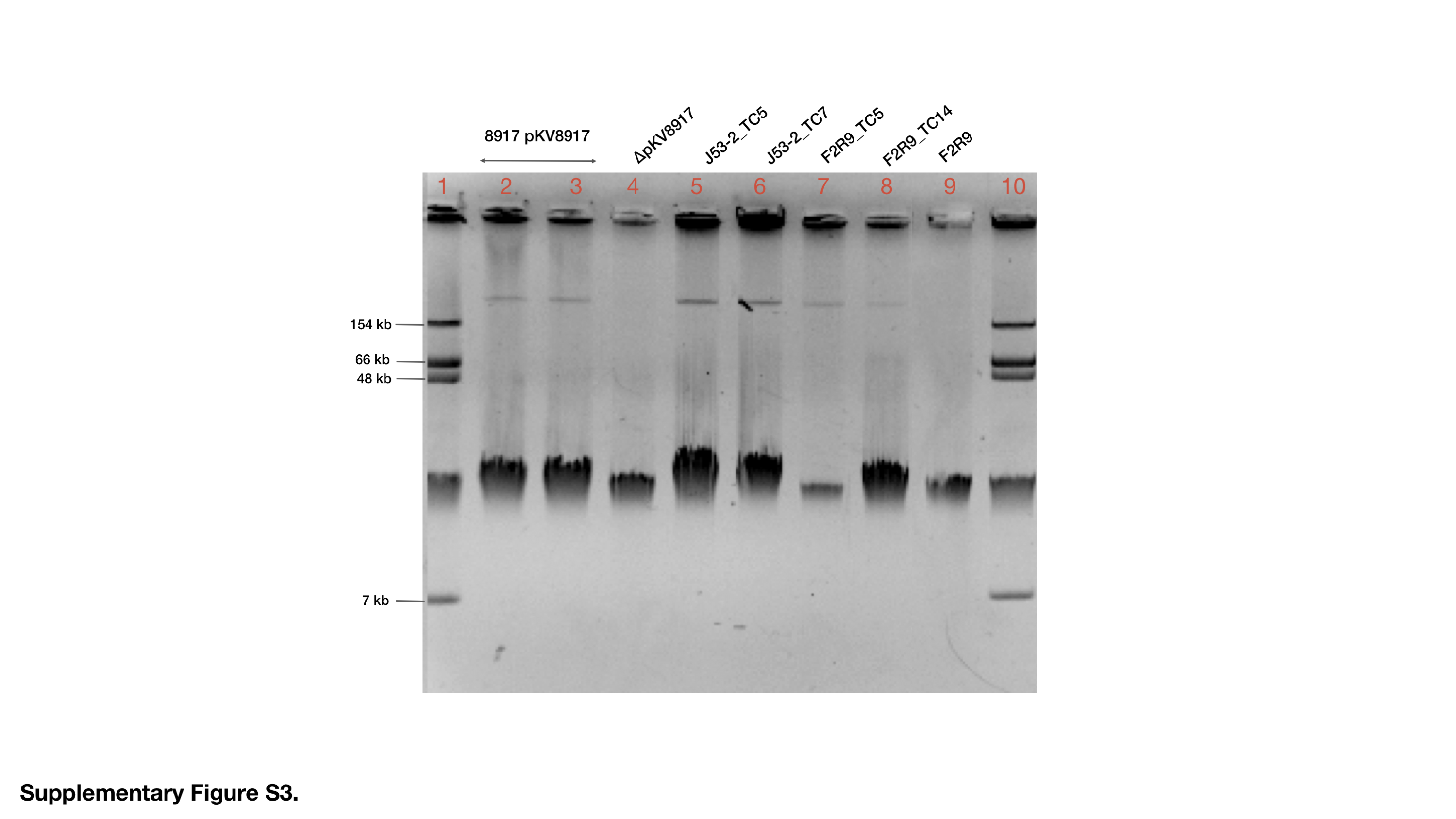

Supplement: Supplementary Figure 3 — Plasmid profile based on alkaline lysis. Lines 1 and 10, E. coli NCTC 50192 plasmids were used as molecular size markers (154-, 66-, 48-, and 7-kb; Bachman et al., 2015); lines 2-3, K. variicola 8917 (pKV8917); line 4, ΔpKV8917 (cured strain); lines 5-6, J53-2_TC5 and J53-2_TC7; lines 7-8, F2R9_TC5 and F2R9_TC14; and line 9, F2R9. [file Image_3.TIFF]

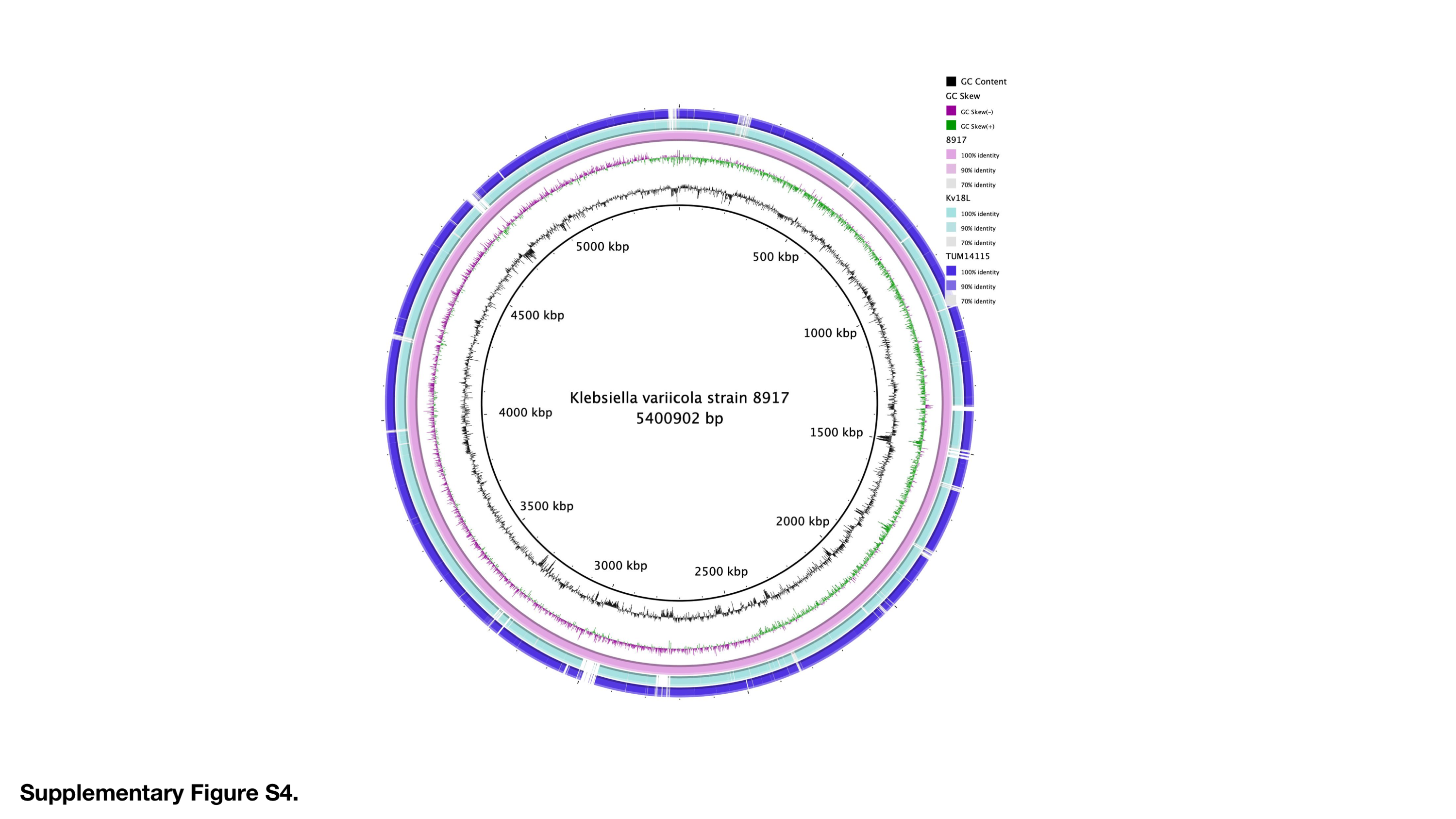

Supplement: Supplementary Figure 4 — Genome sequence alignment of 8917 vs TUM14115 and Kv18L obtained from hmv K. variicola isolates. [file Image_4.TIFF]

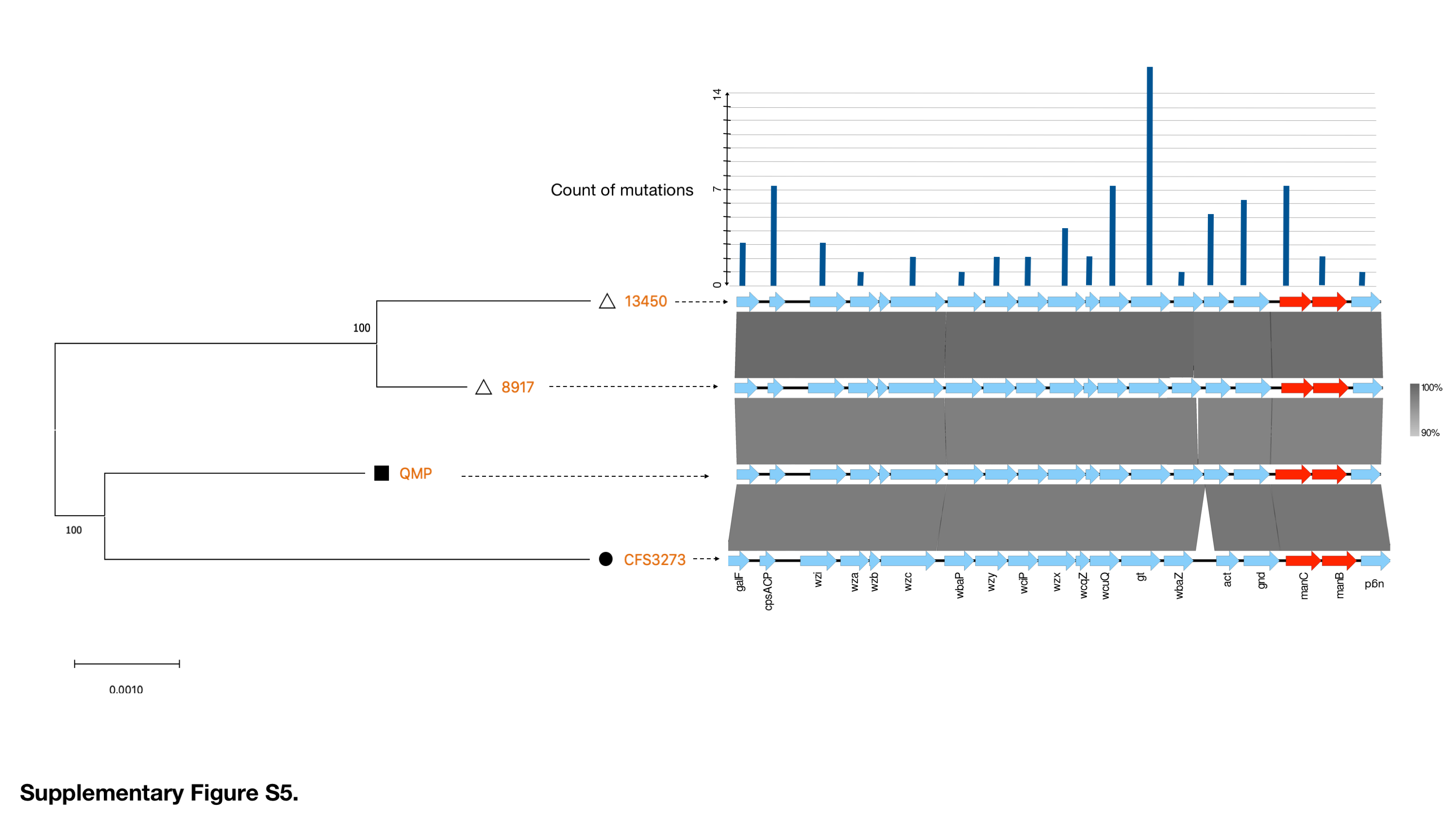

Supplement: Supplementary Figure 5 — Phylogeny of isolates with capsule-type KL114. The phylogenetic tree was based on the concatenated amino acid sequence of 19 proteins that make up KL114 capsular type. K. variicola strains 13450 and 8917 are indicted with a triangle, K. pneumoniae strain QMP with black square and E. coli strain CFS3273 with black circle is showed. In addition, the comparison of the complete KL114 capsule locus. CDSs are presented as arrows. Gray bars indicate regions of similarity, and darker regions indicate a higher degree of similarity. Red arrows correspond to mannose synthesis and processing proteins (ManC and ManB). The number of mutations found across the cps is represented with blue bars. Detailed information on the sites of mutation is in Supplementary Table 3. [file Image_5.TIFF]

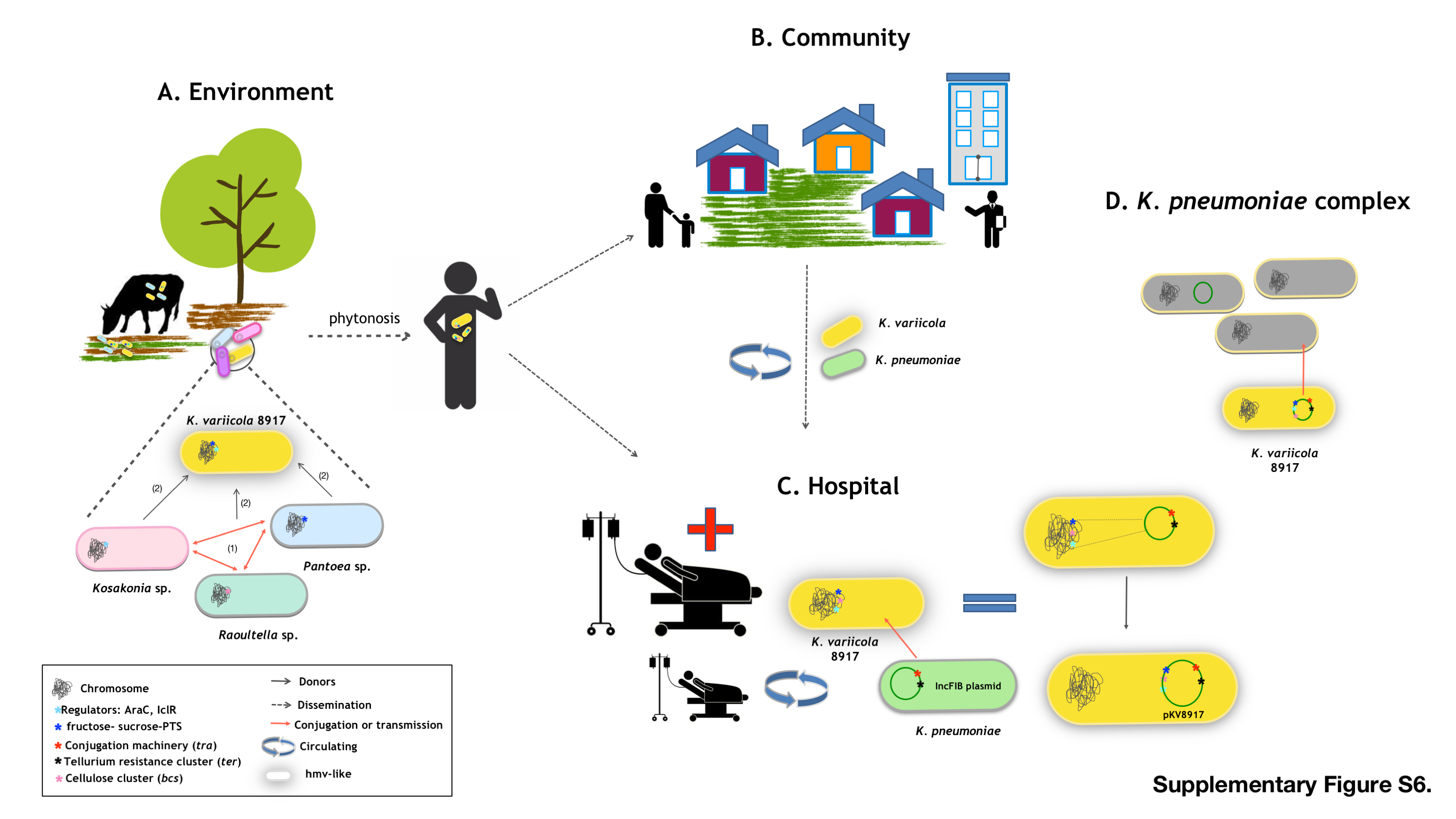

Supplement: Supplementary Figure 6 — A model for how the pKV8917 may have originated. (A) K. variicola is ubiquitous in soil, plants, animals and water. These natural niches possess a diverse bacterial population and allow K. variicola to coexist with other endophytes and bacteria of animal carriage. Pantoea sp. Kosakonia sp. and Raoultella sp. were donors of genetics regions to K. variicola 8917 possibly by horizontal transfer. Raoultella sp. is isolated more frequently from animal gut and sewage so that K. variicola 8917 might have acquired the cellulose biosynthesis cluster (bcs) in this spot. Our hypothesis is that hypermucoviscosity was acquired by bacterial species from the environment. By phytonoses, humans become infected by K. variicola 8917 which could be transported to the community (B) or hospital setting (C) in this scenario K. variicola coexist with K. pneumoniae. IncFIB plasmids (IncFIBp) are usually associated with multidrug-resistant and virulent strains; IncFIBp carries the transference module (tra) and tellurium resistance operon (ter), IncFIBp was transferred by means of conjugation to hmv-like K. variicola 8917. IncFIBp serves as the backbone for the incorporation of the genetic elements acquired from environmental strains resulting in the structure pKV9817. pKV8917 could be transfer the hmv-like to species of the K. pneumoniae complex (D) but also acquire virulence and multidrug-resistance genes. [file Image_6.TIFF]
